# Expanding limits of artificial enzymes: unprecedented catalysis by an oxidase nanozyme in activating a structural protein for covalent crosslinking and conferring remarkable proteolytic resistance†

**DOI:** 10.1039/d4sc03767g

**Published:** 2024-08-20

**Authors:** Adarsh P. Fatrekar, Rasmi V. Morajkar, Amit A. Vernekar

**Affiliations:** a Inorganic and Physical Chemistry Laboratory, CSIR-Central Leather Research Institute Chennai-600020 Tamil Nadu India amitvernekar@clri.res.in; b Academy of Scientific and Innovative Research (AcSIR) Ghaziabad-201002 India

## Abstract

Nature has endowed us with some complex enzymes capable of utilizing proteins as their substrates to generate functional proteins through post-translational modification. However, nanozymes' interplay with proteins as substrates is scarce, with their chemistry predominantly established using only small molecule substrates, featuring a significant gap in this area. Due to the huge prospects of nanozymes in biotechnological and therapeutic interventions, studies establishing the unexplored roles of nanozymes in the biological environments and their interplay beyond small molecule substrates warrant immediate attention. In this study, we unveil the unprecedented role of a Mn-based oxidase nanozyme (MnN) in activating a structural protein, collagen, and covalently crosslinking its tyrosine residues with only a trace amount of tannic acid (TA) without compromising its triple-helical structural integrity. While therapeutic applications demand materials prepared from collagen, the current chemical and physical crosslinking of collagen often presents significant challenges such as toxicity, denaturation, or high costs. MnN lucidly accomplishes crosslinking interplay at its 101 facets using oxygen as a co-substrate under mild conditions. This process takes advantage of MnN being active at mild acidic pH where collagen preferentially exists as a soluble triple helix (monomeric form), exposing functionalities and enhancing the crosslinking degree. Importantly, this reaction also confers 100% resistance to collagenase attack on the collagen tendon-derived biological material. The catalyzed TA–tyrosine linkage in the telopeptide region of collagen probably impedes the initial recognition step of collagenase, providing robust protection against its degradative action. Our study not only expands the repertoire of nanozymes' substrates beyond the existing library of small molecules but also establishes a significant step toward designing a gold standard for collagen crosslinking. With biomedical applications demanding biomaterials derived from protein scaffolds with preserved structural integrity, our investigation bridges the gap between nanozymes' chemistry and crosslinking proteins, opening exciting prospects for biomaterial development.

## Abstract 2

New properties of nanozymes are vital for their advancement. We reveal an oxidase nanozyme's unique role in activating tyrosyl residues for collagen crosslinking with tannic acid, imparting 100% collagenase resistance to crosslinked collagen tendons.

## Graphical abstract

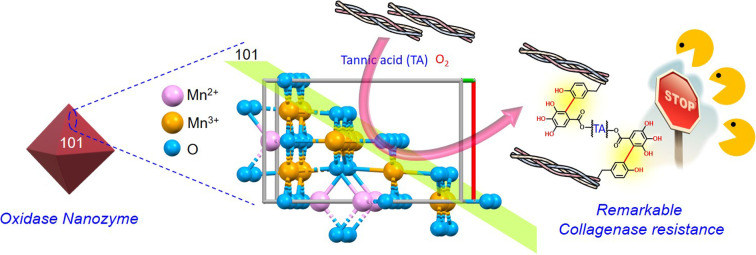

## Introduction

Enzymes with intricate structures are powerful biological catalysts designed by nature. Although they perform their action using a high level of sophisticated architecture surrounding their active site, they still have a lot of limitations. Their susceptibility to denaturation, the high cost of isolation and purification, short shelf life, and inability to achieve expected catalytic efficiency due to sensitivity to reaction conditions other than their native environment, significantly impede their applications.^1,2^ With the advances in research on artificial enzymes, nanomaterial-based enzyme mimetics called nanozymes have attracted significant attention since their inception.^1–4^ They have gained burgeoning interest in replacing enzymes in certain enzyme-based applications due to their advantages such as stability, ease of synthesis, customizability and affordability.^2,3^ Although the lack of selectivity, specificity, and catalytic efficiency of the nanozymes presents notable challenges,^5,6^ there are other significant and unexplored issues that need to be addressed. Several sophisticated enzymes that work with complex mechanisms are known to perform their activity using protein substrates to generate functional proteins or hydrolyzed products.^7–13^ However, reports on nanozymes functioning with protein substrates are very rare and are primarily focused on cytochrome c oxidation and protein hydrolysis.^14–18^ This lack of studies highlights one of the greatest gaps impeding the advancement of nanozymes. It should also be noted that the current applications and chemistry of nanozymes are mostly limited to their role in functioning with small molecule substrates such as colorimetric probes,^19^ reactive oxygen species^20^ and environmental pollutants.^21,22^ While the prospects of nanozymes have been demanding in biotechnological and biomedical interventions, special attention is warranted in studying nanozymes' interplay with complex biomolecular substrates. Understanding their role in handling intricate mechanisms such as protein functionalization and stabilization of proteins through crosslinking is essential for expanding the limits of nanozymes.

**Fig. 1:**
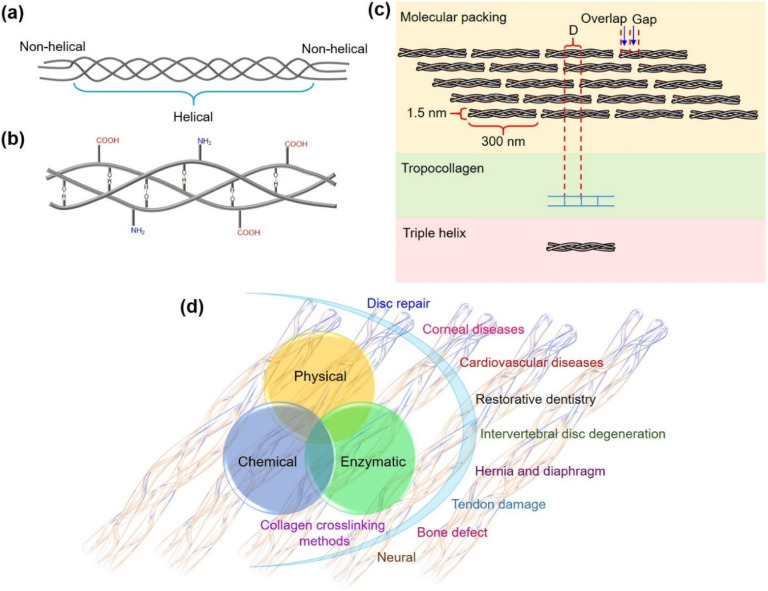
(a) The triple helical structure of collagen. (b) Hydrogen bonding in the triple helix. (c) Schematics showing the assembled collagen molecules in fibrils. (d) Collagen crosslinking methods and the use of crosslinked collagen biomaterials in biomedical applications.

In our efforts on the development of nanozymes and addressing their limitations and opening newer avenues,^23–29^ herein we present the first demonstration of an oxidase nanozyme catalyzing the functionalization and covalent crosslinking of a major structural protein, collagen. Collagen helps in shaping our tissues, ensuring structural integrity and is present in the skin, tendons and bones.^30,31^ The polypeptide chains of collagen assemble together by hydrogen bonding to form a triple helical structure of 300 nm length and 1.5 nm diameter (Fig. 1a and b) that further arranges into fibrils (Fig. 1c).^31^

Biomedical applications set a huge demand for biomaterials prepared from collagen-based scaffolds with conserved structural integrity. The crosslinking of collagen plays a significant role in the development of robust collagen biomaterial that attracts applications in disc repair, cardiovascular diseases, bone defects, tendon damage, corneal diseases, intervertebral disc degeneration, neural damage, and more (Fig. 1d).^32^ However, the use of chemicals such as aldehydes,^33,34^ isocyanates,^35,36^ carbodiimides,^37,38^ photoreactive agents,^39,40^ polyepoxy compounds,^41^ polyphenol^42^ and acyl azides^43^ for crosslinking collagen causes toxicity,^44,45^ calcification,^46,47^ and induces foreign body response,^48,49^ thus seriously limiting their use for biomedical applications. The physical crosslinking methods such as using UV light and dehydrothermal conditions induce conformational changes and cause denaturation of collagen, counteracting the crosslinking ability of UV radiation and making the material susceptible to enzymatic degradation.^32,50–52^

Naturally, collagen is post-translationally modified to achieve crosslinking by lysyl oxidase enzyme during extracellular matrix (ECM) maturation.^53–55^ Lysyl oxidase converts lysyl residues to allysine that further undergoes crosslinking by aldol condensation and Schiff base formation. Furthermore, the transglutaminase enzyme can functionalize collagen by linking it with other proteins to enhance the properties of the resulting structures.^56^ Tyrosinase and laccase enzymes have been explored for crosslinking collagen. During these reactions, the crosslinking of tyrosyl residues of collagen could be enhanced by the external addition of catechol substrates such as caffeic acid, chlorogenic acid and catechin.^57^ Although enzymatic crosslinking provides an upper hand over chemical and physical methods of crosslinking in terms of biocompatibility, it is highly expensive due to the high cost of expression, isolation, purification, storage and recovery of enzymes.^58,59^ Despite active research in this domain, the crosslinking limitations, the complex nature of collagen, and its wide range of applications have projected significant challenges in establishing a gold standard protocol. In our innovative approach, we unravel the unprecedented role of Mn_3_O_4_ octahedral nanoparticles (Mn nanozyme, MnN), a simple nanozyme exhibiting oxidase-like activity while functioning with collagen as a protein substrate. MnN remarkably activates tyrosine residues in the telopeptide region of collagen for covalent crosslinking with only trace amounts of tannic acid (TA) under mild conditions. This process takes advantage of MnN's activity at acidic pH, where collagen preferentially exists as a soluble triple helix (monomeric form), thereby exposing amino acid functionalities and enhancing the crosslinking degree. The crosslinked collagen tendon biomaterial obtained by this approach also exhibits 100% resistance to degradation by collagenase. The MnN-catalyzed linkage of tyrosine residues in the telopeptide region of collagen to TA molecules probably hinders the initial crucial recognition step of collagenase, resulting in robust protection against its degradative action.^60,61^ Our investigation expands the repertoire of nanozymes' substrates beyond the existing library of small molecules and also signifies progress toward advancing strategies for collagen crosslinking. As biomedical applications demand biocompatible materials derived from protein-based scaffolds with preserved structural integrity, our investigation also merges the gap between the chemistry of nanozymes and crosslinking proteins, highlighting new avenues for biomaterial development.

## Results and discussion

### Synthesis and characterization of the oxidase nanozyme

**Fig. 2:**
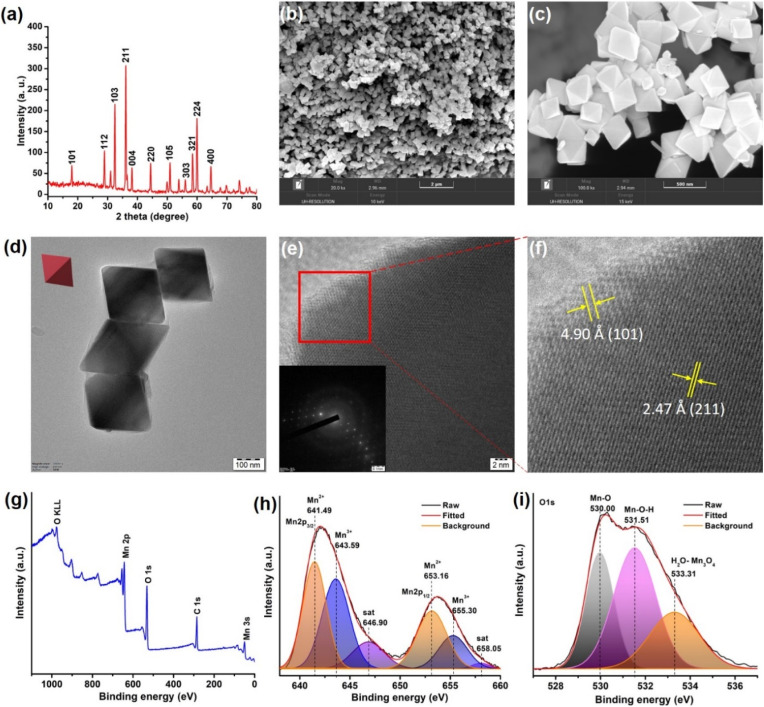
(a) Powder XRD pattern of MnN. (b and c) The low and high-resolution SEM images of MnN showing octahedral nanoparticles, respectively. (d) TEM image of MnN (inset shows a representative red colored octahedron). (e) HRTEM image of MnN showing lattice fringes. Inset shows the SAED pattern exhibiting clear spots due to the crystalline nature of MnN. (f) The magnified portion of the area marked as a red box in panel (e), showing (101) and (211) planes. (g) Survey XPS spectrum of MnN. (h and i) Core-level Mn 2p and O 1s XPS spectra of MnN.

To address the fundamental question of activating collagen, we thought it worthwhile to select a nanomaterial composed of biorelevant Mn ions that could lucidly modulate redox activities.^62^ The use of Mn_3_O_4_ nanoparticles is gaining importance in therapeutics as it offers greater control over redox modulatory activities and can mimic oxidase and multiple antioxidant enzymes.^63,64^ MnN required for the study was synthesized by hydrothermal method, following a previously reported procedure.^65^ The resultant MnN are octahedral-shaped with well-defined edges and surface planes. The synthesized MnN was then characterized by several techniques. The powder X-ray diffraction (XRD) pattern shown in Fig. 2a reveals the appearance of strong-sharp peaks, confirming the crystallinity of the MnN, and corresponding to the tetragonal hausmannite structure. The scanning electron microscopy (SEM) images (Fig. 2b and c) along with transmission electron microscopy (TEM) images (Fig. 2d) of MnN reveal the well-defined crystalline octahedral morphologies having edges of ∼200 nm length. The high-resolution (HR)TEM image (Fig. 2e) recorded at the edge of MnN clearly shows lattice fringes and its inset corresponds to the selected area electron diffraction (SAED) pattern. The appearance of bright spots in the SAED pattern further corroborates with powder XRD data that the particles are crystalline in nature. The magnified image (Fig. 2f) corresponding to the area marked as a red box in Fig. 2e highlights distances of 4.90 Å and 2.47 Å, which are attributed to (101) and (211) planes of MnN octahedral particles, respectively. Categorized in the tetragonal crystal system, the eight exposed facets of MnN octahedral particles are enclosed by {101} planes.^66^

The chemical composition and the valence state of MnN was analyzed by X-ray photoelectron spectroscopy (XPS). The survey XPS spectrum shows peaks characteristic of Mn, C, and O (Fig. 2g). To get further insights into the valence states, the core level Mn 2p_3/2_ and Mn 2p_1/2_ spectra were recorded (Fig. 2h). The deconvoluted Mn 2p spectra show peaks at 641.49 eV and 653.16 eV in Mn 2p_3/2_ and Mn 2p_1/2_, respectively, ascribing to Mn^2+^ ions in MnN. The peaks appearing at 643.59 eV and 655.30 eV in Mn 2p_3/2_ and Mn 2p_1/2_, respectively, are ascribed to Mn^3+^ ions. The satellite signal of Mn 2p appearing at 646.9 eV originates due to the charge transfer from the outer electron shell of the O ligand to the Mn 3d shell. The core-level O 1s XPS spectrum (Fig. 2i) is deconvoluted to result in three peaks. The peak appearing at 530.00 eV is due to Mn–O. Whereas the peak at 531.51 eV suggests hydroxyl groups adsorbed on the surface of MnN, the peak at 533.31 eV relates to the adsorbed H_2_O. These data are in agreement with the previous report.^67^

### Oxidase-like activity and kinetics of MnN

**Fig. 3:**
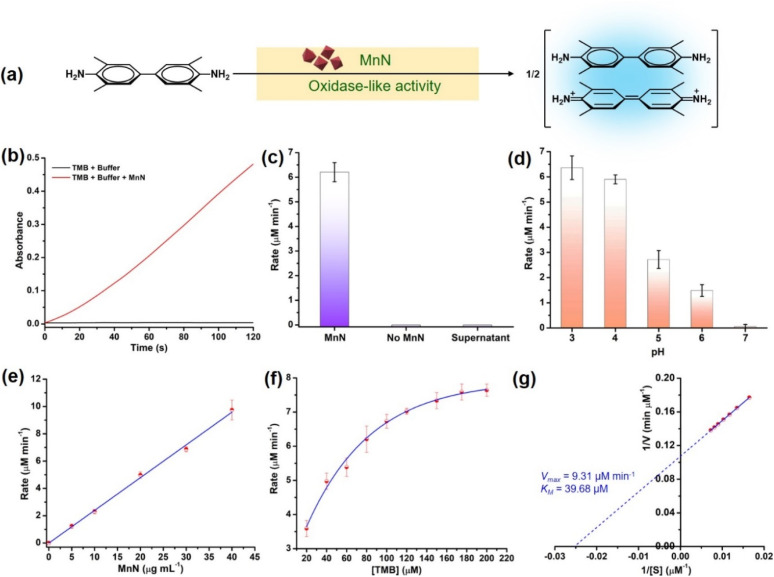
(a) Scheme showing the oxidase-like activity of MnN converting TMB to the oxidized blue-colored charge-transfer adduct that absorbs at 652 nm. (b) Time-dependent monitoring of the formation of oxidized TMB at 652 nm in the presence and absence of MnN. (c) Bar graph showing the comparison of the rate of oxidase-like activity of MnN with other control reactions. (d) Rates of oxidase-like activity at different pH. (e) Effect of increase in the concentration of MnN on its oxidase-like activity. (f) Effect of increase in the concentration of TMB on the oxidase-like activity of MnN, showing Michaelis–Menten kinetics. (g) Lineweaver–Burk plot corresponding to the plot shown in panel (f).

To check the oxidase-like activity, the characterized MnN particles were tested using an oxidase activity assay by employing suitable colorimetric probe substrates. In the presence of MnN, 3,3′,5,5′-tetramethylbenzidine (TMB) was oxidized at pH 4 to the corresponding colored product, absorbing at 652 nm due to the formation of a charge-transfer complex as depicted in Fig. 3a and S1a.† Similarly, other substrates such as *o*-phenylenediamine (OPD) and azino-bis(3-ethylbenzothiazoline-6-sulfonic acid) (ABTS) resulted in orange and green-colored products, absorbing at 450 nm and 420 nm, respectively, as shown in Fig. S1b and c.† The TMB oxidation reaction was monitored in a time-dependent manner (for 120 s) at 652 nm in the presence of 20 μg mL^−1^ MnN which showed the progress of the reaction with an initial rate of 6.20 ± 0.39 μM min^−1^ (Fig. 3b and c). In the absence of MnN, the reaction did not progress (Fig. 3b and c). The supernatant obtained after incubating MnN in water for 1 h, did not show any TMB oxidation (Fig. 3c), confirming that the oxidase-like activity occurs at the surface of MnN.

We further evaluated the oxidase-like activity of MnN at pH ranging from 3–7. It can be seen that the MnN exhibits the highest activity at pH 3 and 4, however, its oxidase-like activity diminishes at higher pH (Fig. 3d and S1d†). The property of MnN being active at acidic pH is highly beneficial for reactions with collagen protein as under acidic conditions, collagen exists exclusively as a soluble triple helix (monomeric form) (*vide infra*).^68–70^ To test whether the oxidase-like activity is dependent on the concentration of MnN, we varied the amount of MnN from 0–40 μg mL^−1^ in the reaction. A proportional increase in the rate of the reaction was observed upon increasing the amount of MnN, which is attributed to the first-order kinetics (Fig. 3e and S1e†). In another experiment, when the concentration of the TMB substrate was varied from 20–200 μM, the initial rate increased proportionally till 80 μM and then started saturating at a higher concentration of TMB (Fig. 3f and S1f†), indicating the rate of formation of MnN–substrate complex equals the rate of decomposition of MnN–product complex. The initial rates obtained using different concentrations of TMB were better fitted to Michaelis–Menten curve as observed for enzyme-catalyzed reactions (Fig. 3f). The parameters such as velocity maximum (*V*_max_) and the Michaelis constant (*K*_M_) were calculated to be 9.31 μM min^−1^ and 39.68 μM, respectively, from the corresponding Lineweaver–Burk plot (Fig. 3g). A low *K*_M_ value indicates a high affinity of MnN towards TMB.

### MnN-catalyzed l-tyrosine and TA activation and crosslinking

l-Tyrosine is a crucial amino acid that has multifaceted biological significance in facilitating molecular recognition. It also serves as a crucial component in the production of neurotransmitters, thyroid hormone, and melanin pigment, and is useful for cognitive function.^71–74^ Tyrosine also forms the basis for numerous vital biological redox processes.^75^ The tyrosine molecules can link together to form dityrosine catalyzed by the dual oxidases (DUOX).^76,77^ The dityrosine crosslinks play a vital role in the design of functional biomaterials having notable applications in tissue engineering.^78,79^ Although lysyl oxidase plays an instrumental role in modifying lysyl residues and crosslinking collagen,^55^ the tyrosine residues present in the telopeptide region also hold potential in the milieu of collagen crosslinking and stabilization. Inspired by the crosslinking abilities of DUOX, we considered it worthwhile to explore the potential of MnN for converting tyrosine to dityrosine. This investigation not only holds potential for collagen crosslinking but also opens up advanced functions of nanozymes in handling large protein substrates.

**Fig. 4:**
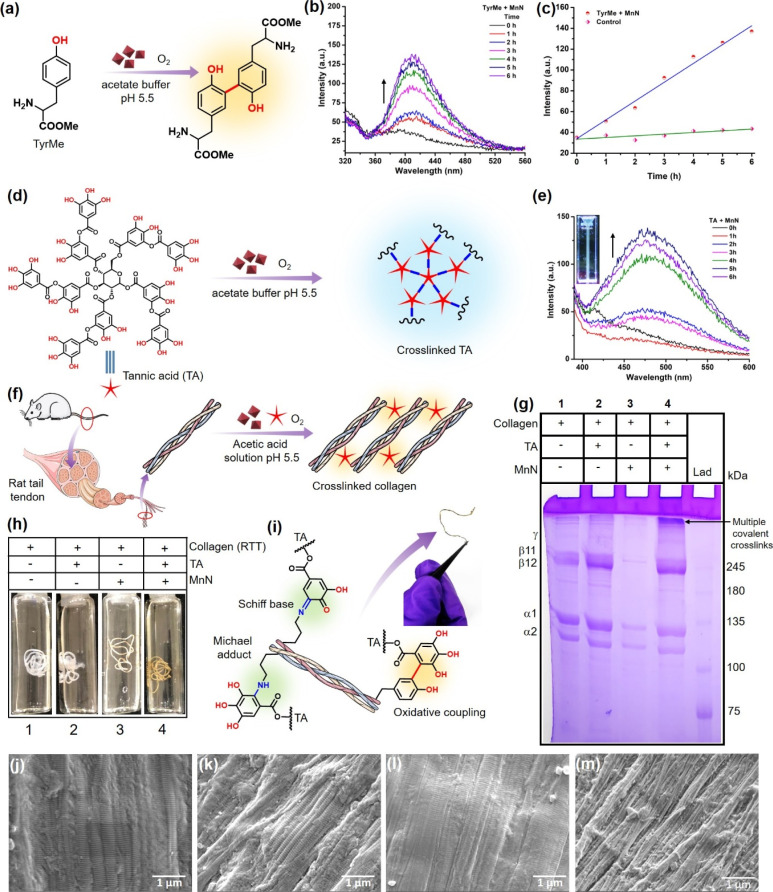
(a) Conversion of TyrMe to its dimer as a result of oxidase-like activity of MnN. (b) Fluorescence spectra showing a time-dependent increase in the intensity of fluorescence due to the continuous formation of TyrMe dimer during the reaction. (c) The line graph corresponding to the fluorescence spectra presented in panel (b). (d) Coupling of TA with TA itself as a result of oxidase-like activity of MnN. (e) Fluorescence spectra showing time-dependent increase in the intensity of fluorescence due to the continuous formation of the TA–TA species during the reaction. Inset shows a cuvette containing the product that exhibits bluish fluorescence when excited at 365 nm. (f) MnN-catalyzed crosslinking of RTT collagen in the presence of TA. (g) SDS PAGE analysis of the crosslinked collagen and other controls, LAD = ladder. (h) Representative photographs of control RTTs and experimental RTT obtained after MnN-catalyzed crosslinking. (i) The possible modes of covalent crosslinking of collagen in addition to the oxidative coupling of phenolics. Inset shows a dried crosslinked RTT that was found to be stable after 12 months of storage. SEM image of (j) RTT, (k) RTT + TA, (l) RTT + MnN and (m) RTT + TA + MnN, after treatment at pH 5.5 for 24 h.

The tyrosine crosslinking ability was assessed by the addition of MnN to the acetate buffer solution containing l-tyrosine methyl ester (TyrMe) (Fig. 4a), which is easily soluble. The formation of dityrosine methyl ester was probed by exciting the reaction mixture at 300 nm and monitoring the characteristic fluorescence peak appearing at 410 nm (Fig. 4b) in a time-dependent manner using a spectrofluorometer.^80^ It can be seen that the intensity of the peak at 410 nm increases linearly with increasing time due to the continuous formation of dityrosine in the presence of MnN (Fig. 4c). In contrast, the control reaction did not show any noticeable formation of dityrosine even for an extended duration (Fig. S2† and 4c). Further, we evaluated the capabilities of MnN for catalytically crosslinking dipeptide and tripeptide, separately, through the formation of dityrosyl links. When MnN was added to l-tyrosyl-l-phenylalanine and l-valyl-l-tyrosyl-l-valine containing solutions in acetate buffer, we observed the formation of crosslinked peptides as probed following a similar method discussed above (Fig. S3a, b and S4a, b†). In the absence of MnN, the peptides did not crosslink (Fig. S3c and S4c†). To the best of our knowledge, this is the first investigation showing an oxidase nanozyme that can indeed convert tyrosine to dityrosine and crosslink peptides using molecular oxygen, thus holding potential for designing biomaterials.

The above results of monophenolics activation by MnN prompted us to investigate the activation and crosslinking of polyphenols like TA. This innovative strategy would also aid in coupling tyrosine units of collagen with TA to result in a covalently crosslinked collagen biomaterial (*vide infra*). To probe this reaction, we added MnN to the acetate buffer (pH 5.5) solution containing TA (Fig. 4d). We monitored the reaction by UV-vis spectroscopy and the formation of coupled polyphenolics was confirmed by spectrofluorometry. It can be seen from Fig. S5a† the appearance of a shoulder signal at 365 nm, which could be ascribed to either the formation of the corresponding quinone of TA or TA–TA coupled products. To confirm if TA–TA species are formed in the reaction, we excited the reaction mixture at 365 nm, which resulted in the appearance of a peak at 475 nm (Fig. 4e). The inset of Fig. 4e shows a cuvette containing the product that exhibits bluish fluorescence when excited at 365 nm. While the coupled products result in the emission spectra, the quinone formed does not fluoresce at this wavelength. This is due to the facile intersystem crossing of the excited quinones to the triplet state, quenching its fluorescence emission.^81^ Several ellagitannin-based natural products containing C–C coupled phenolics, oligodopamine and polydopamine representing C–C coupled phenolics are known to absorb in 350–380 nm region and exhibit emission at ∼450 nm.^82–87^ It should also be mentioned that polytannic acid having such moieties is also known to exhibit fluorescence at ∼450 nm when excited at 360 nm.^84^ In contrast, the other control reactions with TA alone in the absence of MnN, did not result in the formation of quinone and coupled products under our experimental conditions as observed by the absence of corresponding signals in their absorbance and emission spectra (Fig. S5b and S6a†).

### Evaluation of the MnN-catalyzed collagen crosslinking and examining structural integrity

To extend the above findings to collagen crosslinking, we added MnN to the rat tail tendon (RTT) collagen solution (20 mM acetic acid) in the presence of TA (Fig. 4f) at 37 °C and incubated for 24 h. To obtain efficiently covalent-crosslinked collagen biomaterial, processing collagen in its monomeric form is highly essential rather than its fibril form which generally reduces the exposure of functionalities for crosslinking. As monomeric collagen molecules are stable under mildly acidic conditions, developing processes that work under these conditions are imperative. Interestingly, MnN possesses the unique ability to activate and link TA and tyrosyl radicals under mildly acidic conditions, which are otherwise difficult to activate without an oxidase-like nanozyme under these conditions. Thus, it is expected that MnN can covalently crosslink collagen efficiently in its monomeric form. SDS PAGE analysis (Fig. 4g) of the obtained reaction mixture clearly indicates the formation of multiple covalent crosslinks of collagen at the top portion of the resolving gel (lane 4). The bands appear dense and darker probably due to the formation of high molecular weight polymeric collagen structures. It can also be seen that the bands corresponding to β11 and β12 appear darker due to intra-chain crosslinks. Concurrently, the fading of bands accounting for α1 and α2 chains in lane 4 probably suggest that due to the formation of covalent crosslinks and higher molecular weight structures, the concentration of monomeric collagen is diminished. In contrast, the degree of covalent crosslink formation in lanes 1 and 2 for collagen alone and collagen + TA, respectively, was too low as the bands at the corresponding top portions appear faded. This is probably due to the presence of very low levels of crosslinks formed naturally due to the action of the lysyl oxidase enzyme in the RTT. The other bands in the lane 1 and 2 appear darker which probably indicates the absence of covalent crosslinks. Although it is known that TA can form multiple hydrogen bonds with collagen and stabilize it, the SDS PAGE analysis shows that such hydrogen bonds are weaker and are perturbed during analysis. Moreover, the dynamic microenvironment of tissues can perturb hydrogen bond interactions between collagen and TA, resulting in the release of TA that can alter the activities of other crucial enzymes such as acetylcholinesterase (AChE), fatty acid synthase (FAS) and NADPH-CYP reductase. In this milieu, MnN-catalyzed crosslinking of collagen may help in locking TA to the collagen biomaterial and preventing its release. The appearance of faded bands in lane 3 could be due to the adsorption of collagen on MnN which forms a precipitate that cannot be efficiently loaded on the gel. Apparently, the {101} planes of MnN form a template for the adsorption of collagen. However, in the absence of the other reactant, *i.e.*, TA, the reaction does not proceed forward, keeping the collagen adsorbed on the surface of MnN. In contrast, the degree of formation of such precipitate is significantly lower when MnN, collagen, and TA are present together, thus facilitating the release of crosslinked products from the surface of MnN.

While in the above experiments, TA is shown to crosslink collagen, it was crucial to evaluate the crosslinking ability of MnN using other relevant small molecule phenolic substrates like gallic acid (GA). The treatment of GA with collagen in the presence of MnN led to highly crosslinked collagen that could not penetrate the polyacrylamide gel during SDS-PAGE analysis (Fig. S6b†). In another set of experiments, we explored the crosslinking ability of MnN using an alternate protein, BSA, and its analysis by SDS PAGE. Functionalization of BSA by TA molecules and the formation of aggregates of different higher molecular weights suggested that MnN has the capability of crosslinking BSA (Fig. S6c†).

To understand whether the collagen maintains its structural integrity after MnN-catalyzed crosslinking in the presence of TA, we recorded the circular dichroism (CD) spectra (Fig. S7†). Collagen exhibits a positive and a negative band at 223 nm and 198 nm, respectively, attributed to the triple helical conformation (Fig. S7†).^88^ Incubation of collagen with MnN and TA mixture resulted in a very negligible decrease in the molar ellipticity of the band at 223 nm. The increase in the molar ellipticity at 198 nm is probably due to the aggregation of collagen molecules as a result of crosslinking. Similar observations have been previously reported for the interaction of collagen with glutaraldehyde, formaldehyde, Cr(iii), and glycoproteins, which are known to aggregate collagen molecules.^89–91^ Under our experimental conditions and control reactions, the spectral features are indicative of the conservation of triple helical conformation of crosslinked collagen. In contrast, the partial denaturation of collagen has representative signatures of low-intensity signals and red-shifted crossover points.^89^ However, such characteristics are not seen in the spectra presented here. Our investigation of using a nanozyme highlights that preserving the triple helical structure of collagen-based biomaterial during their development is critical for achieving the intended biomedical functions of those materials.

### MnN-catalyzed covalent crosslinking of RTT to confer stability and its analysis

We further extended our work to evaluate the efficacy of MnN in direct crosslinking and stabilizing RTT (Fig. 4h and S8a, b†) to prepare a collagen-based biomaterial. It can be seen from vial 1 shown in Fig. 4h that the RTT alone incubated in the acetic acid solution of pH 5.5 revealed a slight swelling due to microstructural alteration that decreased the fibril packing density.^92^ In the other control tests performed by the incubation of RTT in the presence of a range of concentration of TA (10, 20, 50, 80, 125 and 150 μM), the swelling was observed (vial 2 shown in Fig. 4h and complete panel of Fig. S8a†). This indicates that at such low concentrations of TA without MnN, the hydrogen bonding-assisted crosslinking of RTT could not resist swelling. In contrast, it is interesting to note that in the presence of MnN and TA at different concentrations as mentioned above, RTT was found to be intact (vial 4 shown in Fig. 4h and complete panel of Fig. S8b†). This observation probably suggests that the covalent C–C bond-assisted crosslinking of RTT strongly prevents the decrease of the fibril packing density. In the absence of TA, MnN also exerts some swelling preventive properties (vial 3 shown in Fig. 4h).

It is known that under aqueous mild acidic conditions, keto-moieties strongly prefer to form imines/Schiff bases with amines or Michael adducts.^93,94^ Therefore, the possibility of the formation of Schiff bases/Michael adducts from quinone (produced from MnN-catalyzed reaction) and lysyl residues of collagen under mildly acidic conditions may not be ruled out. The formation of such covalent adducts along with tyrosyl residue coupling is represented in Fig. 4i (a photograph of the crosslinked RTT biomaterial is also shown in Fig. 4i). To understand the formation of these moieties, we modeled the reaction using 3,5-di-*tert*-butylcatechol (DTBC) and *n*-butylamine in the presence of MnN (Fig. S9a†). To stabilize and detect the formation of any Schiff bases as they are prone to hydrolysis, NaBH_4_ was added in the second step of the reaction to obtain corresponding secondary amine. The mass spectrum shown in Fig. S9b† confirms the formation of adducts. The formation of quinone (Fig. S10a†) from DTBC was probed by UV-vis spectroscopy (Fig. S10b†). The appearance of a peak at 420 nm accounts for the corresponding quinone of DTBC.^95^ In addition to these studies, the crosslinked RTT was analyzed by XPS (Fig. S11†). The C 1s XPS has been deconvoluted into peaks accounting for C–C, C–NH, C=N and C=O. The appearance of a peak at 286.31 eV (ref. 96 and 97) in control RTT accounts for the naturally formed imine due to the action of the lysyl oxidase enzyme (Fig. S11a†). However, the increase in the area and the relative intensity of the C=N peak (Fig. S11b†) in the MnN-catalyzed crosslinked RTT, as compared to the control, are indicative of the formation of additional Schiff bases from the reaction of lysyl residues of collagen and TA. These experiments suggest that the additional crosslinking of RTT due to the formation of Schiff bases and Michael adducts may lead to stable structural integrity while maintaining the packing density of collagen molecules.

To analyze the morphological changes of RTT after MnN-catalyzed crosslinking, the RTTs were imaged by SEM (Fig. 4j–m). Whereas the control RTT shows the fibrils and its D-band periodicity (Fig. 4j), the RTT-treated with TA alone shows partial deposition of TA over the fibrils (Fig. 4k). Similarly, MnN-treated RTT also shows the fibrils with their corresponding D-band periodicity (Fig. 4l). In contrast to these three observations, the RTT treated with MnN and TA shows a well-intact, protected and densely packed bunch of fibrils (Fig. 4m). This appearance is probably due to the catalytic action of MnN in the presence of TA that thwarts swelling.

### Evaluation of the collagenolytic stability of the MnN-catalyzed covalently crosslinked RTT tendons

**Fig. 5:**
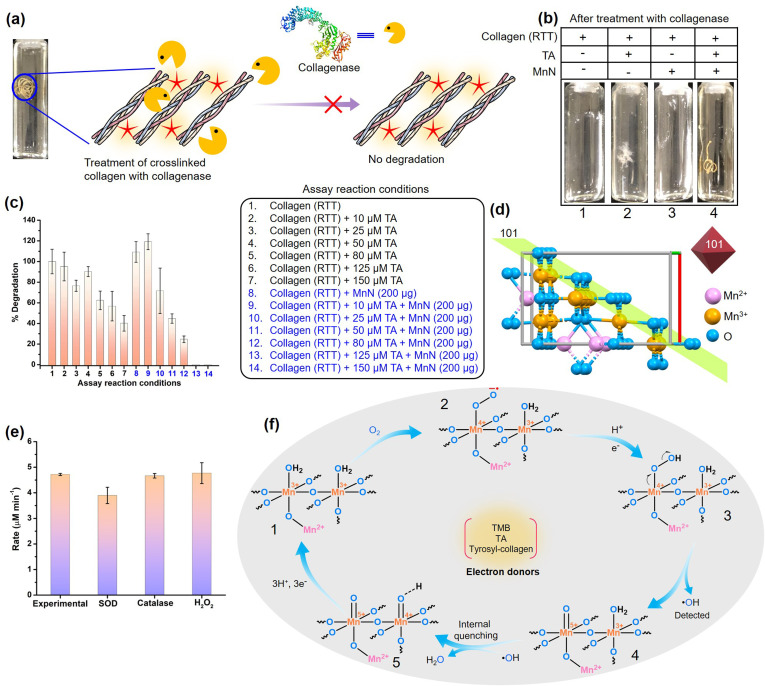
(a) Scheme showing treatment of the crosslinked RTT with collagenase. (b) Photographs of the RTTs obtained after collagenase treatment. Note that RTTs were destroyed in control reactions. However, the MnN-catalyzed crosslinked RTT is intact. (c) Comparison of % degradation of RTTs by collagenases as analyzed by hydroxyproline estimation assay. (d) The crystal structure of MnN showing the arrangement of ions at the exposed 101 planes of the octahedral nanoparticle. The structure is made using a cif file of Mn_3_O_4_ (ID: mp-18759) provided in the materials project database. (e) Probing oxidase mechanism by studying the effect of using reactive oxygen species scavenging enzymes such as SOD and catalase, and the addition of H_2_O_2_ on the oxidase-like activity of MnN. (f) The proposed mechanism by which the molecular oxygen is converted to water as a result of the transfer of electrons from electron donors such as TMB, TA or tyrosyl residues of collagen.

Noting the fact that collagen exhibits superior biocompatibility, but limiting other favorable biomaterial characteristics, efficient crosslinkers are important for the production of robust collagen-based biomaterials that are strongly resistant to collagenases. However, the absence of benchmark crosslinking methods impedes the biomedical application of collagen-based biomaterials. To understand the collagenolytic stability of the crosslinked RTT obtained by the action of MnN and TA, we incubated the crosslinked RTT and other control RTTs with collagenase under standard reaction conditions (Fig. 5a and S12†). We observed that the RTTs from the control reactions, *i.e.*, RTT alone, RTTs treated with a range of TA concentrations (10, 25, 50, 80, 125, 150 μM), and RTT with MnN were degraded by collagenase within 24 h (vials 1–3 in Fig. 5b and S12a†). While RTTs treated with lower TA concentrations were susceptible to collagenase degradation, even those treated with 125 and 150 μM TA were not highly resistant, which may be due to TA hydrogen bonded to collagen. Although under these conditions some inhibitory effects over collagenase degradation are seen, it is important to mention that the structures of the tendons were significantly disrupted by collagenase (Fig. 5b and S12a†). Interestingly, it was observed that the MnN-catalyzed crosslinked RTT remained intact against the degradative action of collagenase with an increase in the concentration of TA (vial 4 of Fig. 5b and S12b†). At TA concentration as low as 125 μM, the MnN-treated RTT was found to be totally intact. To analyze the extent of degradation, we estimated hydroxyproline content from the reaction media by separating the solid insoluble mass (Fig. 5c). While at the highest control TA concentration, *i.e.*, 150 μM (assay condition 7), 40% degradation of RTT was observed, it is commendable to mention that MnN was able to impart 100% protection against collagenase degradation at TA concentration as low as 125 μM (assay condition 13). To the best of our knowledge, this property is remarkable for a collagen-based biomaterial. Furthermore, we observed that this RTT was intact even after 48 h of collagenase treatment. In the absence of TA, MnN alone has no role in the protection of RTT. As free TA molecules have efficiency in inhibiting collagenase, the released TA, if any, can inhibit collagenase activity. Therefore, we tested collagenase obtained from the primary experimental reaction mixture for its activity on fresh RTT, and we observed 100% degradation. This indicates that the covalently crosslinked TA molecules (phenolics C–C coupled or Schiff and Michael adduct linked to collagen) are not leached into the reaction medium, unlike only hydrogen-bonded TA molecules. This property is also crucial as the other vital enzymes such as AChE, FAS and NADPH-CYP reductase should not be compromised during the biomaterial's application.

Due to the complexity of collagen structure, we speculate that the excellent protective effects from collagenase observed in the MnN-catalyzed crosslinked RTT probably lie in hindering in the mechanism of collagenase action. Collagenases are known to cleave collagen at a defined site, typically Gly–Leu/Ile, resulting in ¼ and ¾ length fragments through a complex molecular mechanism.^60^ As per mechanistic findings, they primarily proteolyze the recognition sites from the C-terminal telopeptide on the outer surface of the collagen fibril without directly impacting the actual cleavage site.^60,61^ The hydrolysis of the C-terminal telopeptide region exposes the cleavage site to facilitate triple helix unwinding, collagenolysis and trigger interactions of collagenase with adjacent tropocollagen molecules.^60,61^ Interestingly, MnN facilitates the coupling of TA with the tyrosyl residues found in the telopeptide region of tropocollagen (α1 chain of collagen type I from rat has three tyrosyl residues in the C-terminal telopeptide region). Moreover, one lysyl residue is also present in the C-telopeptide region of α1 chain of collagen type I. This lysyl residue along with the majority of lysyl residues in the triple helix region can take part in the formation of a Schiff base or Michael adduct. Collectively, we believe that these covalent modifications occurring at the primary collagenase recognition site probably interfere in the initial recognition mechanism that likely prevents collagenase attack. Moreover, prevention of swelling of RTT further restricts collagenase's access to recognition and binding sites of collagen fibril. It is also important to mention that Mn_3_O_4_ has been recently shown to protect cartilage by detoxifying reactive oxygen species, thereby having the potential for osteoarthritis therapy.^98^ Mn_3_O_4_ inherently does not alter the expression of collagen and matrix metalloproteinases. Even under oxidative stress conditions, the redox modulatory properties of Mn_3_O_4_ successfully aided in restoring the appropriate expression levels of collagen and matrix metalloproteinases.^98^

### Mechanistic insights into the oxidase-like activity of MnN and its crosslinking activity

Although MnN shows steady-state kinetics or enzyme-like mechanism in the oxidative conversion of TMB, it is crucial to probe reactions occurring at the surface of MnN to get deeper insights into how the tyrosyl residues from the telopeptide region of collagen are activated for crosslinking. As MnN belongs to the tetragonal crystal system, the eight exposed facets of MnN octahedral particles are enclosed by {101} planes on the surface which provide a template for the reaction to occur. These planes are composed of multiple O-linked Mn^3+^ sites while Mn^2+^ resides in the interior bulk (Fig. 5d). To understand whether dissolved molecular oxygen plays a role in catalysis, we checked TMB oxidation under oxygen-deprived conditions (0.5 ppm O_2_) and pure O_2_ purged conditions (25.3 ppm O_2_). Interestingly, the rate of the TMB oxidation reaction decreased under oxygen-deprived conditions as shown in Fig. S13a,† confirming that O_2_ molecules are necessary and they act as electron acceptors. The rate of the reaction under pure O_2_ purged conditions was found to be similar to that observed for normal air conditions (9 ppm O_2_), indicating that dissolved O_2_ under this condition is sufficient for the high oxidase-like activity of MnN. O_2_ reduction may lead to the generation of partially reduced or completely reduced oxygen species such as superoxide anion radical (O_2_˙^−^)/H_2_O_2_ or water, respectively. We probed the generation of O_2_˙^−^ as a result of single electron reduction using a standard formazan production assay (Fig. S13b†). However, the absence of any peak at 570 nm accounting for formazan in the UV-vis spectrum confirmed that the possibility of single electron reduction may be ruled out. This was further confirmed by the addition of superoxide dismutase (SOD) enzyme in the reaction mixture which led to no change in the rate of TMB oxidation (Fig. 5e). To understand whether the reaction proceeds by a two-electron reduction pathway to generate H_2_O_2_ that may show additional oxidation activity, we added catalase enzyme in the reaction mixture to scavenge H_2_O_2_, if generated. As the reaction rate remained unaffected under this condition (Fig. 5e), we confirmed that free H_2_O_2_ was not produced during the catalysis. In another assay, when we added H_2_O_2_ externally to the reaction mixture, the rate was found to be similar to the experimental reaction (Fig. 5e). This indicates that even if H_2_O_2_ molecules are generated, they may not participate in the reaction as MnN performs the oxidase reaction with selectivity towards O_2_. Interestingly, we observed a marginal production of hydroxyl radical (˙OH) by a fluorescence assay (Fig. S13c†). The extremely low levels of ˙OH species suggest the probable role of adjacent Mn^3+^ ions that might quench them, generating H_2_O. As TMB acts as an electron donor, it is expected that tyrosyl residues and TA rings can indeed give out an electron each at the MnN interface, producing tyrosyl and TA radicals as shown in Fig. S14.† The tyrosyl radicals from multiple collagen molecules and TA radicals then can couple together to form crosslinks. In an overall proposed mechanism (Fig. 5f), the MnN-catalyzed reactions constitute the activation of O_2_ at {101} planes to generate the superoxide-bound intermediate 2 with one electron oxidation of Mn^3+^. The protonation step and an electron transfer from the substrates (TMB, TA or tyrosyl residues of collagen) follows next to generate a corresponding Mn-bound hydroperoxide as an intermediate 3 that concomitantly produces ˙OH and results in the Mn-oxo intermediate 4. The generated ˙OH radicals are scavenged and converted to H_2_O by the adjacent Mn^3+^ ions, producing the intermediate 5. This step is followed by successive electron transfers from the substrates, thus completing the cycle. The generation of similar metal-bound reactive intermediates like hydroperoxide and oxo occurs in Mn-based systems during electron transfer electrocatalysis.^99,100^

## Conclusions

Nature's potential in designing sophisticated enzymes that can function with other proteins as substrates is intriguing. On the other hand, nanozymes' chemistry is established only on the basis of their function with small molecule substrates. With the evergrowing use of nanozymes for biotechnological and nanomedicine applications, studies that establish nanozymes' interplay with proteins are urgently required to expand the research domain of artificial enzymes. In this context, we have shown for the first time that oxidase nanozyme can indeed function with biomacromolecules, including peptides and proteins, as part of their new substrate repertoire. We have unraveled the role of an Mn-based oxidase nanozyme that activates the structural protein, collagen, for covalent crosslinking by tethering tannic acid to the telopeptide region under mild reaction conditions. Importantly, this strategy remarkably conserves the self-assembled triple helical integrity of collagen and confers 100% resistance to collagen tendons from collagenase attack, which is probably due to the modification of the telopeptide region, thus hampering the collagenase's initial recognition mechanism. The implications of our investigation are broad, offering a new strategy to address significant challenges in fields utilizing collagen biomaterials, such as disc repair, cardiovascular diseases, bone defects, and more. This investigation not only highlights a significant step toward establishing a gold standard in collagen crosslinking but also opens new avenues for the application of nanozymes in the domain of biomedical science. Furthermore, this work also highlights that oxidase nanozymes can find useful applications in the synthesis of medicinal drugs containing intramolecular dityrosine units. Our research effectively bridges the gap between nanozymes' chemistry and protein crosslinking, unveiling new opportunities for advancing the development of biological materials.

## Author contributions

APF and AAV conceived the idea. APF synthesized and characterized the nanozyme, and performed the kinetics and experimental work with collagen. RVM assisted in the experimental work with collagen and helped in establishing the mechanism. AAV supervised the project. All the authors wrote and edited the manuscript.

## Conflicts of interest

There are no conflicts to declare.

## Supplementary Material

SC-015-D4SC03767G-s001

## Data Availability

The data will be available from the corresponding author on request.
