# Supplementary material for: Expanding limits of artificial enzymes: unprecedented catalysis by an oxidase nanozyme in activating a structural protein for covalent crosslinking and conferring remarkable proteolytic resistance
Source: Chem Sci. 2024 Aug 20;15(36):14726–38. doi: 10.1039/d4sc03767g (PMC11337028; doi:10.1039/d4sc03767g)
Supplement: SC-015-D4SC03767G-s001 [file SC-015-D4SC03767G-s001.pdf]

Supplementary Information (SI) for Chemical Science.
This journal is © The Royal Society of Chemistry 2024

Supporting Information

Expanding limits of artificial enzymes: unprecedented catalysis by an oxidase nanozyme in activating structural protein for covalent crosslinking and conferring remarkable proteolytic resistance

*Adarsh P. Fatrekar, Rasmi V. Morajkar, and Amit A. Vernekar**

A. P. Fatrekar, R. V. Morajkar, A. Vernekar
Inorganic and Physical Chemistry Laboratory
Council of Scientific and Industrial Research (CSIR)-Central Leather Research Institute (CLRI)
Chennai - 600020, Tamil Nadu, India
E-mail: amitvernekar@clri.res.in

A. P. Fatrekar, R. V. Morajkar, A. Vernekar
Academy of Scientific and Innovative Research (AcSIR),
Ghaziabad – 201002, India

Chemicals

Nitric acid 69%, hydrogen peroxide (H_2O_2) solution 30%, catalase from bovine liver, superoxide dismutase bovine, terephthalic acid, sodium hydroxide (NaOH), L-tyrosine methyl ester (TyrMe) 98%, Tannic acid (TA), 2-methoxyethanol, acrylamide, sodium dodecyl sulfate, ammonium persulfate, *N,N,N′,N′*-tetramethylethylenediamine, 2-mercaptoethanol, bromophenol blue were purchased from Sigma-Aldrich chemicals Pvt. Ltd. potassium permanganate, 99.5% ($KMnO_4$), EDTA disodium salt dihydrate, 99.5%, acetic acid glacial extrapure, 99.9%, 3,3,5,5-tetramethylbenzidine dihydrochloride anhydrous, 99% (TMB), *o*-phenylenediamine free base, 99% (OPD), 2,2-azino-bis(3-ethylbenzothiazoline-6-sulfonic acid) diammonium Salt, 98.5% (ABTS), sodium acetate trihydrate, 99.5%, citric acid

monohydrate extrapure, 99.7%, sodium citrate tribasic dihydrate, extrapure, 99%, acetone extrapure, 99.5%, thiazolyl blue tetrazolium bromide (MTT), 98%, sodium phosphate dibasic anhydrous, 99%, sodium phosphate monobasic monohydrate, 99%, n-butylamine, 99%, chloramine-T trihydrate, 98%, *p*-dimethylaminobenzaldehyde, 99%, tris hydrochloride (Tris HCl), glycerol (Glycerine) anhydrous, 99.5 %, bovine serum albumin (BSA), fraction V for molecular biology, 98 %, gallic acid (GA) pure 98% were purchased from SRL chemicals Pvt. Ltd. Collagenase type I from *Clostridium histolyticum* (collagenase) was purchased from Himedia laboratories. 3,5-Di-tert-butylcatechol (DTBC) was purchased from TCI Chemicals (India) Pvt. Ltd.

Characterization

UV-visible spectra were recorded on Shimadzu UV-1800 UV spectrophotometer, scanning electron microscopy (SEM) imaging and X-ray mapping were carried out using Tescan Clara scanning electron microscope. Powder XRD analysis was performed on the Rigaku mini flex-II desktop (Cu Kα 1.5406 Å radiation). X-ray photoelectron spectroscopy (XPS) was recorded on Thermo Scientific, MultiLab 2000 with Al Kα radiation (1486.6 eV) operated at 15 KV. Transmission electron microscopy (TEM) images and selected area electron diffraction (SAED) patterns were recorded on the JEOL JEM 2100 Plus electron microscope by drop-casting finely dispersed sample on a carbon-coated copper grid. Circular dichroism (CD) spectra were recorded on Jasco J-815 CD spectrometer. Mass spectra were recorded on Shimadzu LCMS8040 triple quadrupole mass spectrometer. Sodium dodecyl sulfate-polyacrylamide gel electrophoresis (SDS PAGE) was run using the Bio-Rad PowerPac basic power supply model. Dissolved oxygen was measured using LABWAN bench-type model LWIO-40 dissolved oxygen meter.

1. Synthesis of Mn_3O_4 octahedral nanoparticles (MnN)

MnN was synthesized by hydrothermal method according to the previously reported procedure.[1] Typically, a 4 mL aqueous solution of 0.5 M EDTA disodium salt was dropwise added to the 10 mL aqueous solution of $KMnO_4$ with stirring. The solution was diluted to 70 mL with distilled water and its pH was adjusted to 6.0 by dropwise addition of 2 M HNO_3. The entire solution was then transferred to a 100 mL teflon-lined autoclave reactor and was heated at 180 °C for 6 h. After cooling, the brown precipitate was collected by centrifugation and was washed 6 times with distilled water. Finally, the precipitate was washed once with acetone and was dried in an oven at 70 °C.

2. Oxidase activity, enzyme-like kinetics and factors affecting the activity of MnN

The oxidase activity of MnN was checked by UV-visible spectrophotometry using substrates like TMB, OPD and ABTS. 20 μg MnN was added to a solution of TMB (80 μM), OPD (40 μM), or ABTS (12 μM) in 100 mM, citrate buffer of pH 4. The solution was immediately mixed and its absorbance spectra were recorded. For time-dependent kinetics, the absorbance of this reaction was recorded at 652 nm. A control reaction was performed without the addition of MnN. The enzyme-like kinetics was studied using TMB as a substrate. Factors affecting the rate of the reaction such as pH and leaching of ions were checked. From the plots of time versus absorbance, the rates of the reactions were calculated.

3. Reaction of TyrMe with MnN and detection of dimerized product

1 mM TyrMe was reacted with 200 μg mL^{-1} MnN in 20 mM acetate buffer (pH 5.5) at room temperature (30 ºC) with stirring in the dark. 1 mM TyrMe without MnN under other identical conditions was used as a control. The fluorescence spectra of the reacted solutions were recorded every hour by exciting at 300 nm.

4. Reaction of tyrosine-containing peptides with MnN and detection of crosslinked peptides

1 mM *L*-Tyrosyl-*L*-phenylalanine and 1mM *L*-valyl-*L*-tyrosyl-*L*-valine solutions were separately reacted with 1 mg mL^{-1} MnN in 20 mM acetate buffer of pH 5.5 by stirring in dark at room temperature (30 ºC). 1 mM *L*-Tyrosyl-*L*-phenylalanine and 1 mM *L*-valyl-*L*-tyrosyl-*L*-valine solutions without MnN under other identical conditions were used as controls. The fluorescence spectra of the reacted solutions were recorded by exciting at 300 nm.

5. Extraction of rat tail tendons (RTT) and acid soluble type I collagen

Isolated tails of adult albino male rats were washed with water and stored at -20 ºC. During the extraction of tendons, the tails were thawed and tendons were removed according to the previous report.[2] Acid soluble type I collagen was isolated from the extracted tendons by following the process described earlier.[3] The concentration of extracted collagen solution was found by the hydroxyproline assay.[4]

6. MnN catalyzed TA crosslinking and its detection

100 µM TA was treated with 200 µg mL^{-1} MnN in 20 mM, pH 5.5 acetate buffer at room temperature (30 ºC) with stirring in the dark. The control reaction was performed using TA alone in the absence of MnN in 20 mM acetate buffer (pH 5.5). The absorbance and fluorescence spectra of the reacted solutions were recorded after every hour. To obtain the fluorescence spectra, the samples were excited at 365 nm. The photographs of the reacted solutions were captured by keeping the solutions under 365 nm UV light in quartz cuvettes.

7. MnN catalyzed reaction of TA with acid-solubilized collagen and SDS PAGE analysis

1 µM collagen solution and 200 µM TA were reacted in the presence of 200 µg mL^{-1} MnN were reacted in 10 mM, pH 4.5 acetate buffer (pH range 4-5.5 is beneficial for the reaction) by

shaking at 37 ºC in an incubator. The control reactions were performed using the same concentrations of collagen solution, collagen with TA and collagen with MnN in 10 mM, pH 4.5 acetate buffer. After 24 h, the SDS–PAGE analysis of 20 µL reacted solution (upon treatment with SDS 3X loading dye at 95 ºC for 10 min) was performed using 5% stacking gel and 8% resolving gel. The gel was run at 100 V for 150 minutes. The gel was washed with distilled water and was stained using Coomassie Brilliant Blue according to the previously reported procedure.[5]

8. MnN catalyzed reaction of GA with acid-solubilized collagen and SDS PAGE analysis

1 µM collagen solution and 1 mM GA were reacted in the presence of 200 µg mL-1 MnN in 20 mM acetate buffer (pH 5.5) by stirring at 30 ºC. The control reactions were performed using the same concentrations of collagen solution, collagen with GA, and collagen with MnN in 20 mM acetate buffer (pH 5.5). After 24 h, the reacted solutions were dialyzed against 20 mM acetate buffer (pH 5.5) for 72 h by replacing the dialysis buffer for every 12 h. SDS–PAGE analysis of 20 µL reacted solution (upon treatment with SDS 3X loading dye at 95 ºC for 10 min) was performed using 5% stacking gel and 8% resolving gel. The gel was run at 100 V for 240 min. The gel was washed with distilled water and was stained using Coomassie Brilliant Blue according to the previously reported procedure.[5]

9. MnN catalyzed reaction of TA with BSA and its SDS PAGE analysis

4.5 µM (300 µg mL-1) BSA solution and 400 µM TA were reacted in the presence of 200 µg mL-1 MnN in 20 mM acetate buffer (pH 5.5) by stirring at 30 ºC. The control reactions were performed using the same concentrations of BSA solution, BSA with TA, and BSA with MnN in 20 mM acetate buffer (pH 5.5). After 24 h, the SDS–PAGE of 20 µL reacted solution (upon treatment with SDS 3X loading dye at 95 ºC for 10 min) was performed using 5% stacking gel and 12% resolving gel. The gel was run at 100 V for 240 min. The gel was washed with distilled

water and was stained using Coomassie Brilliant Blue according to the previously reported procedure.[5]

10. MnN catalyzed reaction of TA with acid-solubilized collagen and analysis by CD spectroscopy

1 µM collagen solution and 10 µM TA were reacted in the presence of 50 µg mL^{-1} MnN in 10 mM, pH 5 acetate buffer by shaking at 37 ºC in an incubator. After 6 h, the CD spectra of the reacted solutions were recorded. The control reactions were performed by using the same concentrations of collagen solution, collagen with TA and collagen with MnN in 10 mM acetate buffer (pH 5).

11. MnN catalyzed reaction of RTT with TA

One freshly extracted RTT was added each to the 1 mL solution of 0, 10, 25, 50, 80, 125 and 150 µM TA prepared in pH 5.5 distilled water (adjusted using dilute acetic acid). 200 µg mL^{-1} MnN was added to the solutions and the reaction mixtures were kept on a rotating shaker in the dark at room temperature (30 ºC) for 24 h. The control reactions were performed without the addition of TA or MnN. After 24 h, the RTTs were washed 3 times with distilled water of pH 5.5.

12. MnN catalyzed reaction of DTBC and butylamine

20 mg DTBC and 8.93 µL butylamine (1:1 mole %) were mixed in 2 mL of 1:1, acetonitrile: pH 5 acidified water solution. 2 mg MnN was added to this solution and it was sonicated. The reaction was stirred in the dark at room temperature (30 ºC) for 24 h. 20 mg of $NaBH_4$ was added to the solution after 24 h and it was stirred for 30 min. The products were extracted with ethyl acetate. The solution was dried by evaporating ethyl acetate and to it acetonitrile was

added. Finally, the solution was filtered through a 0.22-micron filter and its ESI-MS was recorded.

13. Reaction of DTBC with MnN and detection of its corresponding quinone

200 μM DTBC was reacted with 20 μg mL^{-1} MnN in 1 mL 20 mM acetate buffer of pH 5 mixed with 0.2 mL acetonitrile solution and its UV-visible spectrum was recorded after 1 h. A control reaction was performed without the addition of MnN.

14. SEM imaging and XPS of RTT treated with TA in the presence of MnN

One freshly extracted RTT was added each to the 1 mL solution of 0, 10, 25, 50, 80, 125 and 150 μM TA prepared in pH 5.5 distilled water (adjusted using dilute acetic acid). 200 μg mL^{-1} MnN from the stock solution was added to it and the reaction was kept on a rotating shaker in the dark at room temperature (30 ºC) for 24 h. The control reactions were performed without the addition of TA or MnN. After 24 h, the RTTs were washed 3 times with pH 5.5 distilled water, dried by lyophilization and directly used for SEM imaging, and XPS analysis.

15. Collagenase treatment of RTT treated with MnN and TA and assessing collagenase resistance by hydroxyproline assay

One freshly extracted RTT was added each to the 1 mL solution of 0, 10, 25, 50, 80, 125, and 150 μM TA prepared in pH 5.5 distilled water (adjusted using dilute acetic acid). 200 μg mL^{-1} MnN from the stock solution was added to it and the reaction was kept on a rotating shaker in the dark at room temperature (30 ºC) for 24 h. The control reactions were performed without the addition of TA or MnN. After 24 h the RTTs were washed 3 times with pH 5.5 distilled water and were dried by lyophilization. 2 mg of lyophilized tendons were separately weighed, and to them, 1 mL of 50 mM tris HCl buffer (pH 7.2) containing 200 μg mL^{-1} collagenase and 5 mM $CaCl_2$ were added. The reactions were placed on a shaking incubator at 37 ºC for 24 h.

The solutions were centrifuged at 13000 rpm for 5 min and 0.5 mL of the clear supernatant was used for hydroxyproline assay. The hydroxyproline assay to find the collagen concentration in the supernatant solution was performed according to the previously reported method.[4] The relative % degradation in each sample was calculated by considering the collagenase degradation of the lyophilized control tendon as 100 %. To check the deactivation of collagenase, to the remaining 0.5 mL clear supernatant a fresh tendon was added and was placed on a shaker in an incubator at 37 ºC for 24 h.

16. Oxidase activity of MnN under normal air, O_2-deprived, and O_2-Saturated conditions

Buffer, TMB and MnN stock solutions were purged with N_2 or O_2 for 1 h. 20 µg MnN from N_2/ O_2 purged stock solution was added to a N_2/ O_2 purged solution of 80 µM TMB in 100 mM citrate buffer (pH 4). The solution was immediately mixed and its time-dependent absorbance was recorded at 652 nm. The reaction under normal air conditions was performed without purging N_2 or O_2.

17. Superoxide anion radical detection experiment using MTT

To the solution of 100 µM TMB prepared in 100 mM pH 4 acetate buffer, 200 µg mL^{-1} MTT and 50 µg mL^{-1} MnN were added. The reaction was stirred in the dark for 1 h and 100 µL of this reaction mixture was added to 900 µL of DMSO, mixed well, and its absorbance spectra were recorded. Control reactions were performed using the same concentrations of MTT, MTT + TMB and MTT + MnN in 100 mM acetate buffer (pH 4).

18. Hydroxyl radical detection using terephthalic acid

1 mg mL^{-1} of MnN was added to 0.5 mM aqueous solution of terephthalic acid whose pH was adjusted to 6.0 by the addition of dilute NaOH. The solution was stirred in the dark. After the

time interval of 1, 3 and 6 h, 1 mL reaction solution was centrifuged and the fluorescence spectra of clear supernatant were recorded by exciting at 315 nm. The emission was checked at 420 nm. The control reaction was performed without the addition of MnN.

19. Oxidase activity of MnN in the presence of SOD, catalase and H_2O_2

20 µg MnN was added to 80 µM TMB solution in 100 mM, citrate buffer of pH 4 containing 1 unit SOD/ 1 unit catalase or 10 mM H_2O_2. The solution was immediately mixed and its time-dependent absorbance was recorded at 652 nm. The control reactions were performed without the addition of SOD/ catalase or H_2O_2. From the plots of time versus absorbance, the rates of the reactions were calculated.

20. Protein images

The image of the collagenase enzyme was created using Chimera software using PDB entry 4ARE.

Note: For proper visualization of plots, the time-dependent monitoring of TMB oxidation has been normalized to a constant initial absorbance.

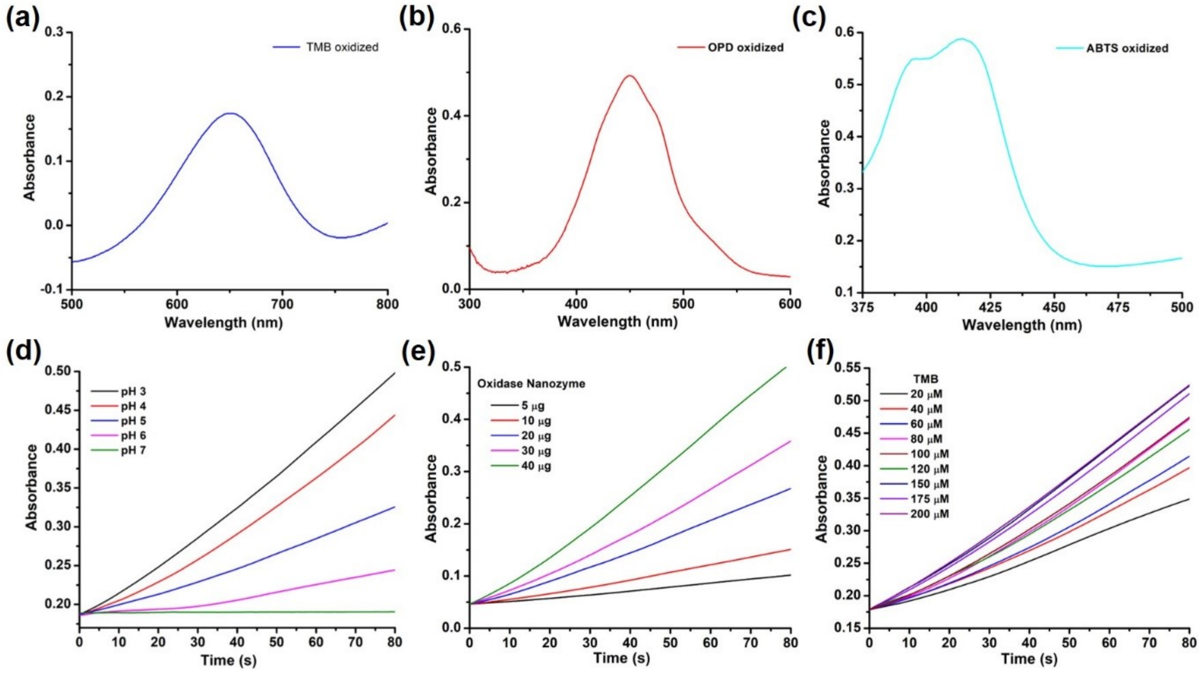

Figure S1. (a-c) UV-vis spectra of oxidized TMB, OPD and ABTS formed as a result of the oxidase-like activity of MnN, respectively. (d-f) Time-dependent monitoring of absorbance of the formation of oxidized TMB catalyzed by MnN under different pH, nanozyme concentration, and TMB concentration conditions, respectively.

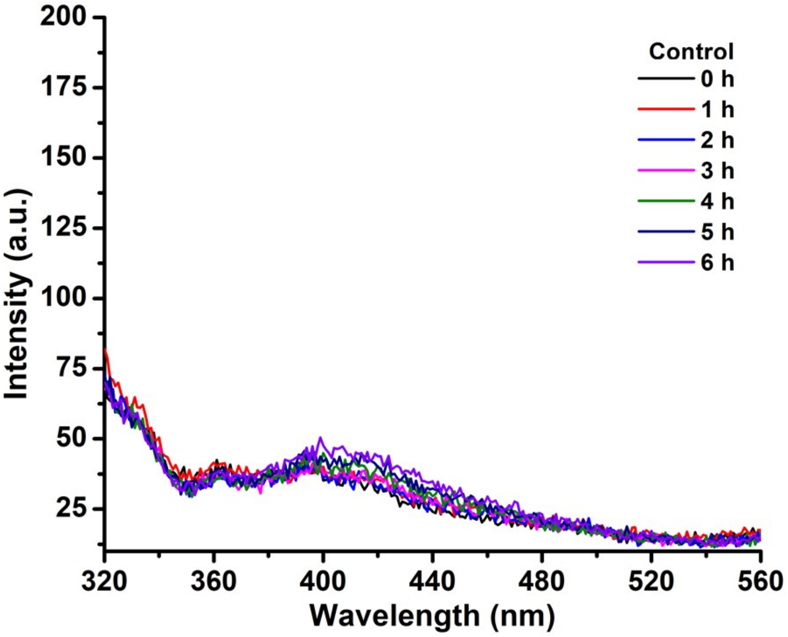

Figure S2. Fluorescence spectra for testing the formation of di-TyrMe in the absence of MnN. Appearance of no signal confirms the absence of dimers.

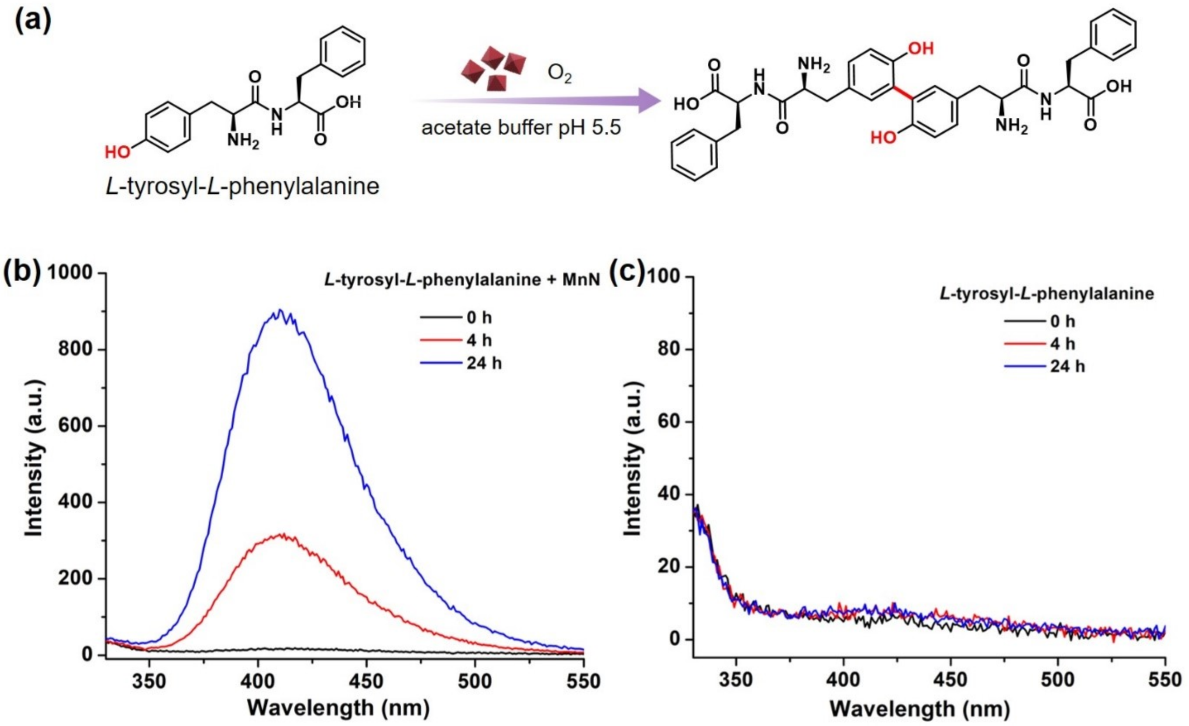

Figure S3. (a) A scheme showing conversion of *L*-tyrosyl-*L*-phenylalanine to its dimer catalyzed by MnN. (b-c) Fluorescence spectra showing the formation of the dipeptide dimer in the presence and absence of MnN, respectively.

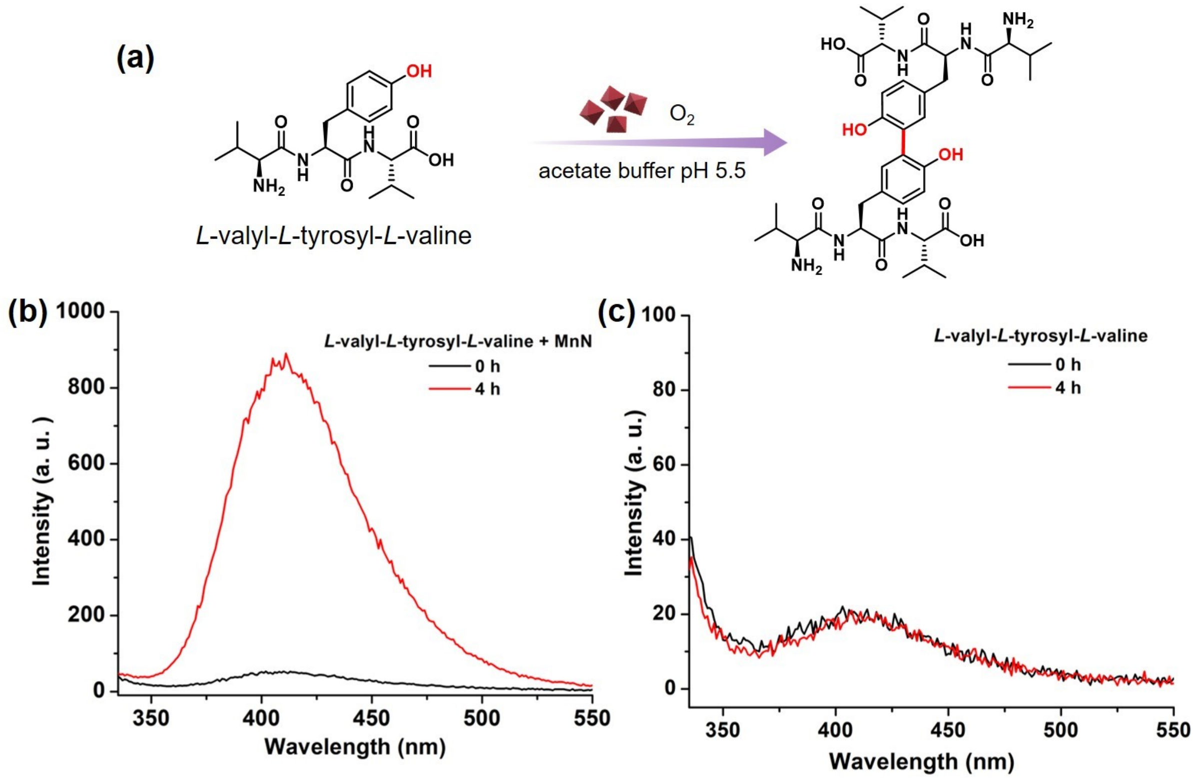

Figure S4. (a) A scheme showing conversion of *L*-valyl-*L*-tyrosyl-*L*-valine to its dimer catalyzed by MnN. (b-c) Fluorescence spectra showing the formation of the tripeptide dimer in the presence and absence of MnN, respectively.

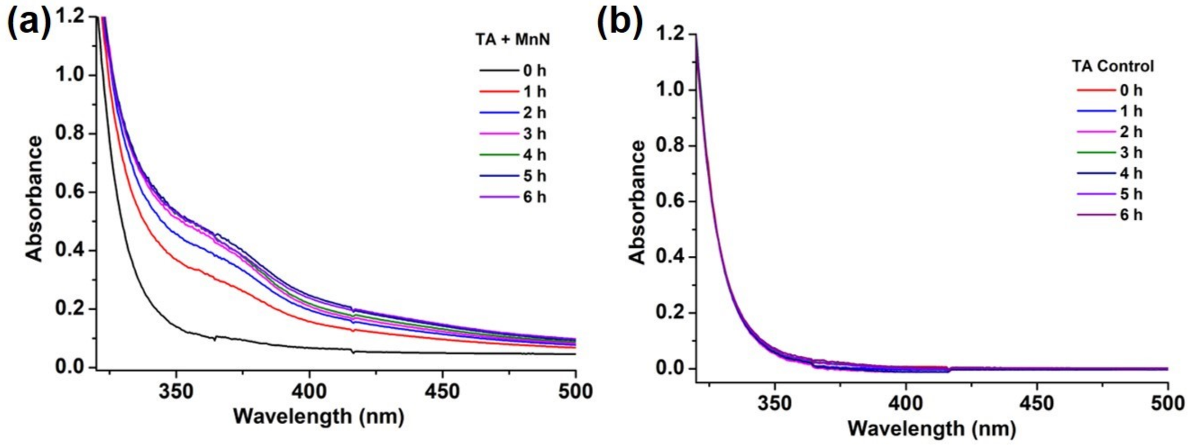

Figure S5. UV-vis spectra of the reaction mixture containing a) TA treated with MnN, b) TA incubated without MnN.

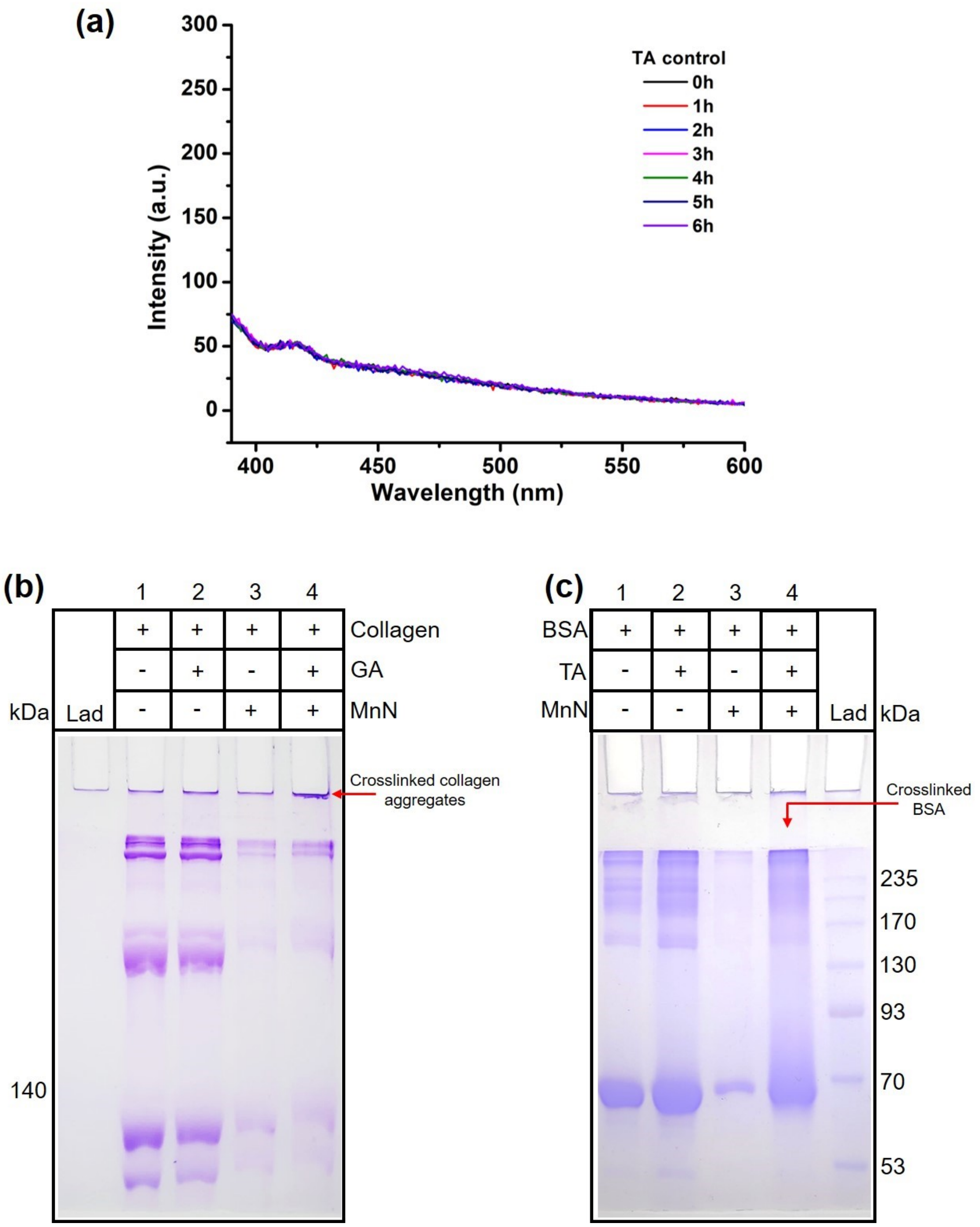

Figure S6. (a) Fluorescence spectra of the control reaction containing TA alone. (b) SDS-PAGE image of collagen crosslinked with GA in the presence of MnN and other representative controls. (c) SDS-PAGE image of BSA crosslinked with TA in the presence of MnN and other representative controls. LAD = Ladder

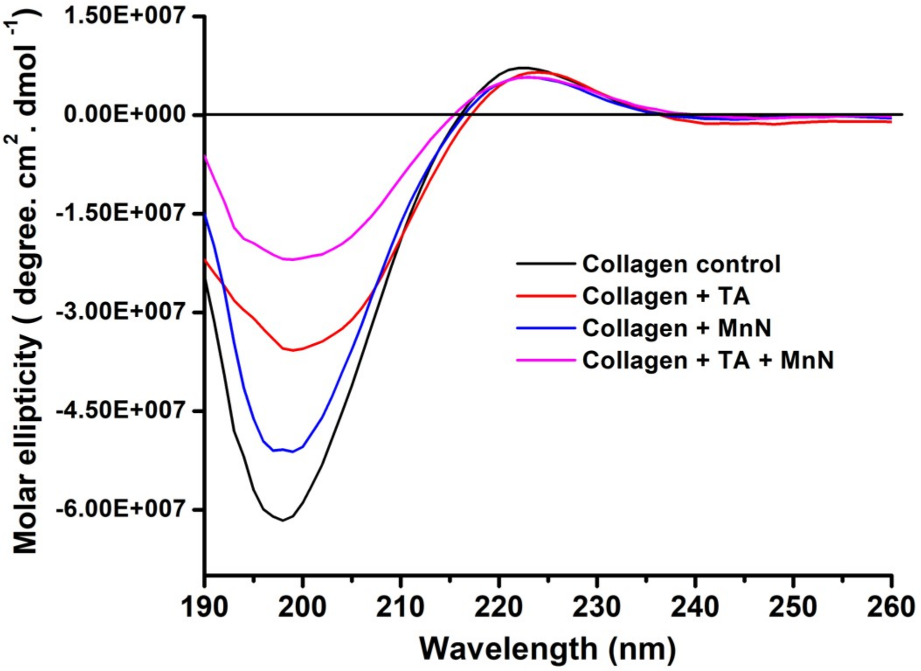

Figure S7. CD spectra of solution containing collagen under various experimental conditions as shown in the legend of the plot.

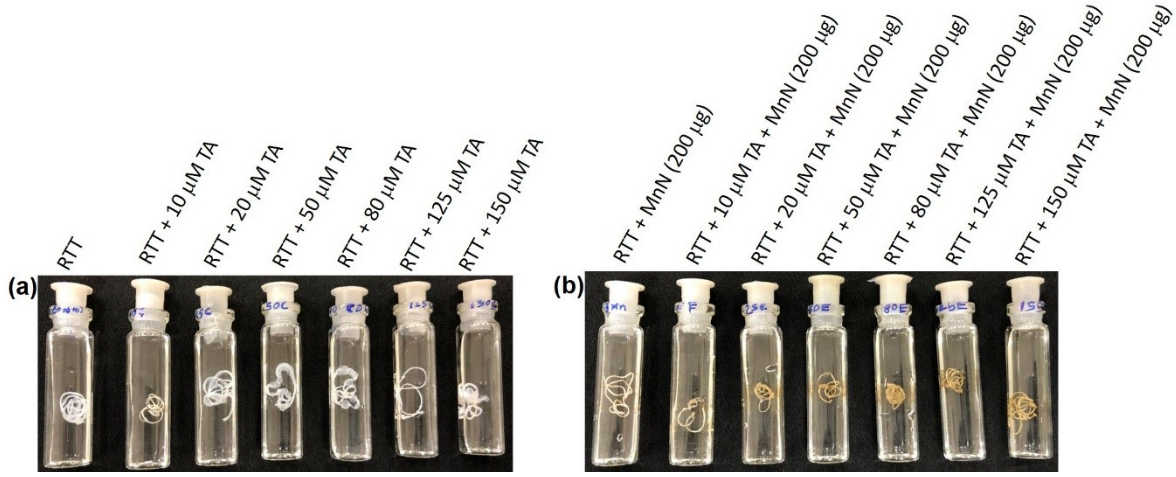

Figure S8. (a) The photographs of vials containing control RTT, and RTTs reacted with different concentrations of TA. Swelling of RTTs can be seen. (b) The photographs of vials containing RTT treated with MnN and RTTs treated with different concentrations of TA in the presence of MnN. The RTTs appear intact.

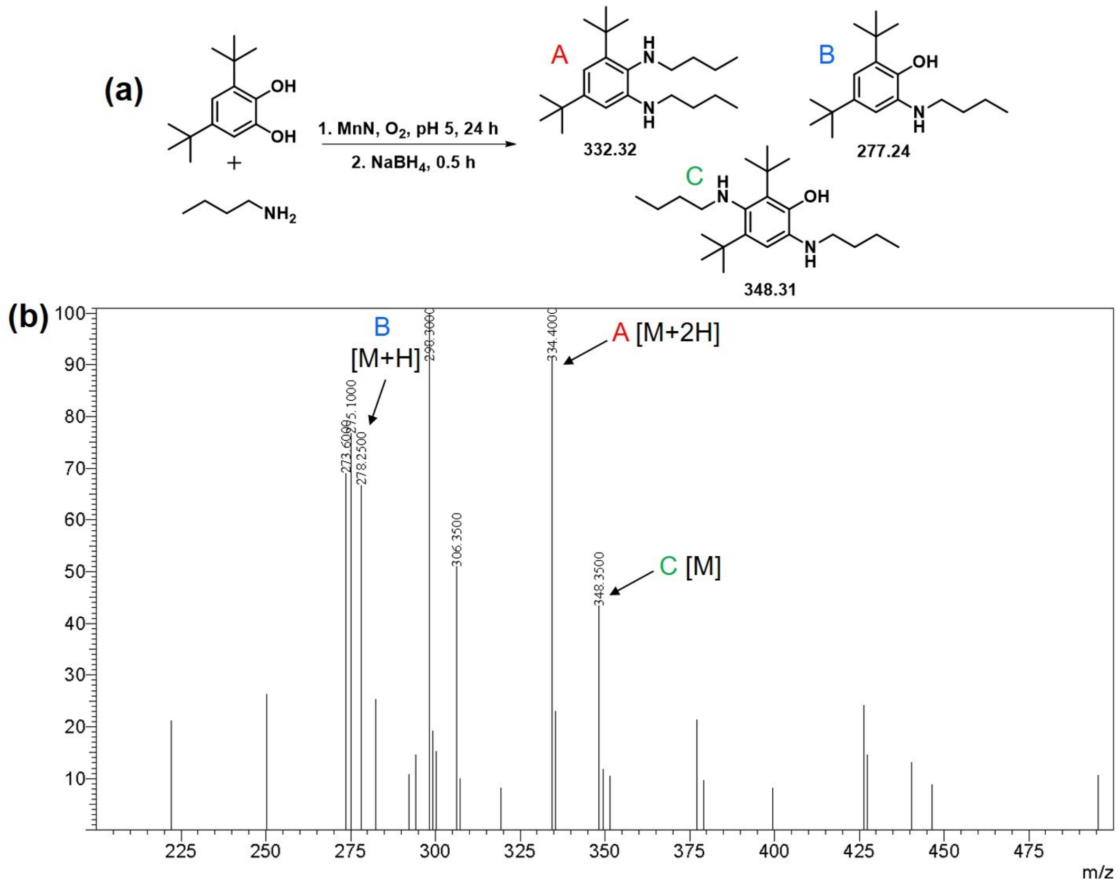

Figure S9. (a) Scheme showing the reaction of DTBC and n-butylamine in the presence of MnN. The DTBC quinone intermediate formed during the reaction reacts with amine resulting in the Schiff base and Michael adduct. Note that $NaBH_4$ was used to reduce Schiff base as it is prone to hydrolysis. (b) Mass spectrum showing the corresponding signals due the formation of adducts.

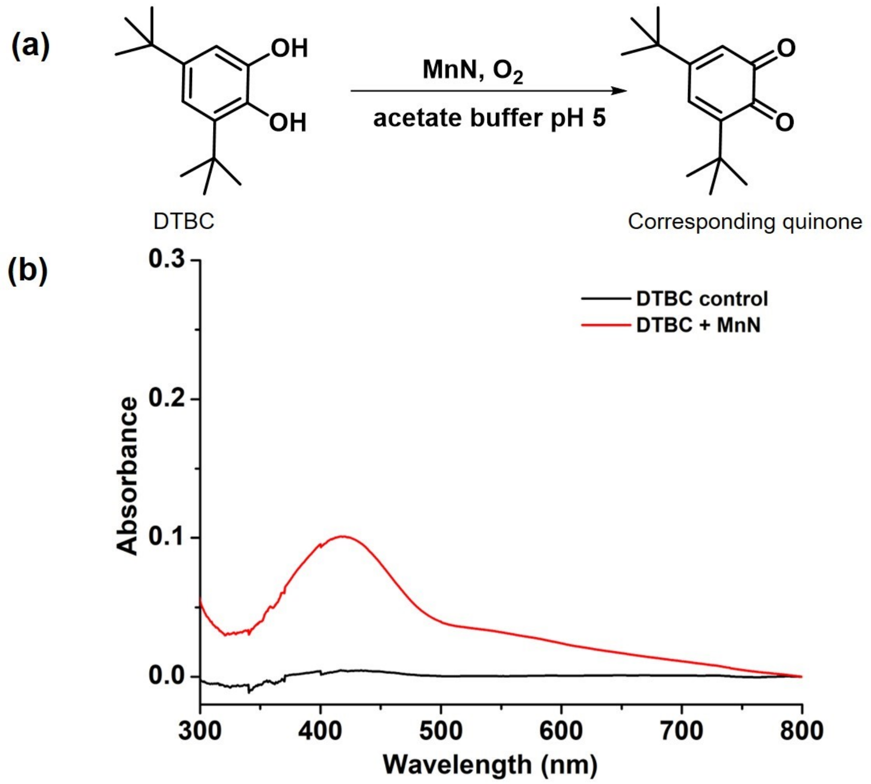

Figure S10. (a) Scheme showing the conversion of DTBC to its quinone due to the oxidase-like activity of MnN. (b) UV-vis spectra showing the peak at 420 nm confirms the formation of DTBC quinone in the presence of MnN. No quinone is formed in the absence of MnN.

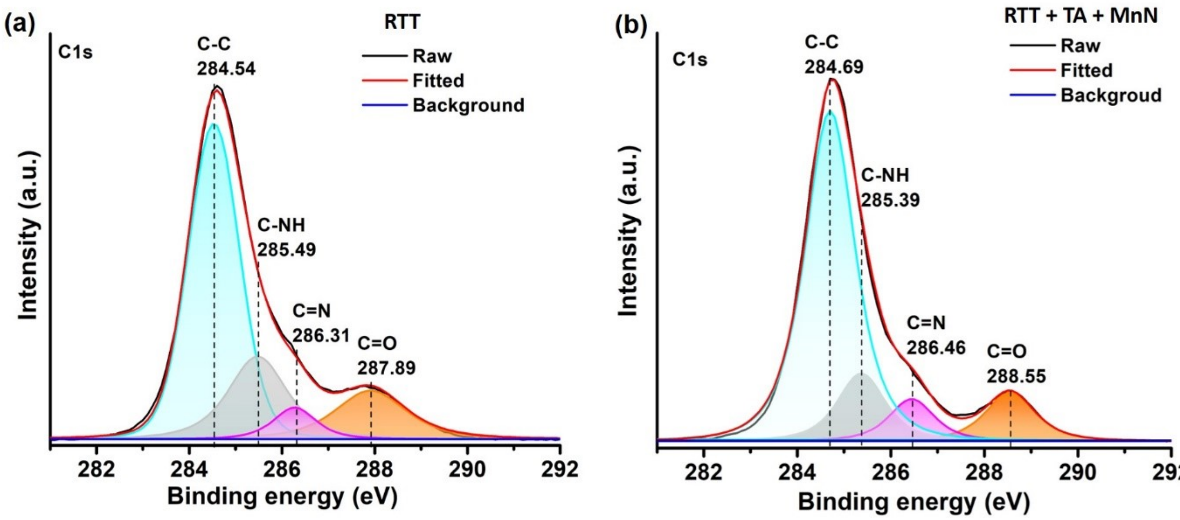

Figure S11. (a-b) C1s XPS spectra of RTT and RTT treated with TA in the presence of MnN. The increased intensity and the area of the peak corresponding to C=N in plot (b) indicate the formation of Schiff bases as a result of oxidase-like activity of MnN.

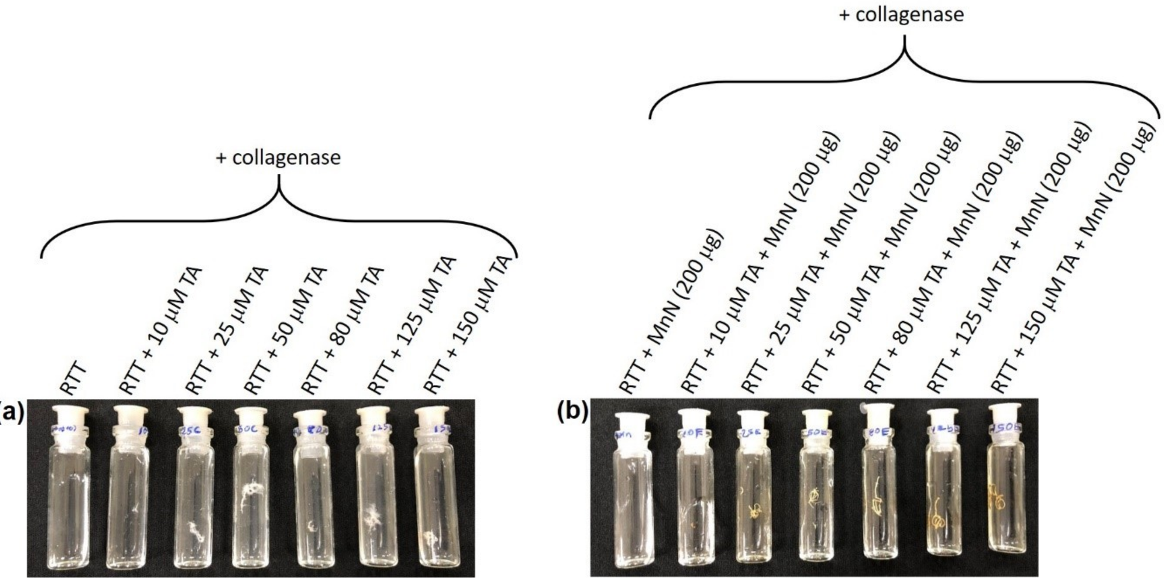

Figure S12. (a) Photographs of vials containing RTTs (obtained after the reaction with different concentrations of TA alone) treated with collagenase showing degradation after 24 h. (b) Photographs of vials containing RTTs (obtained after the reaction with different concentrations of TA and MnN) treated with collagenase showing no degradation at TA concentration as low as 125 μM.

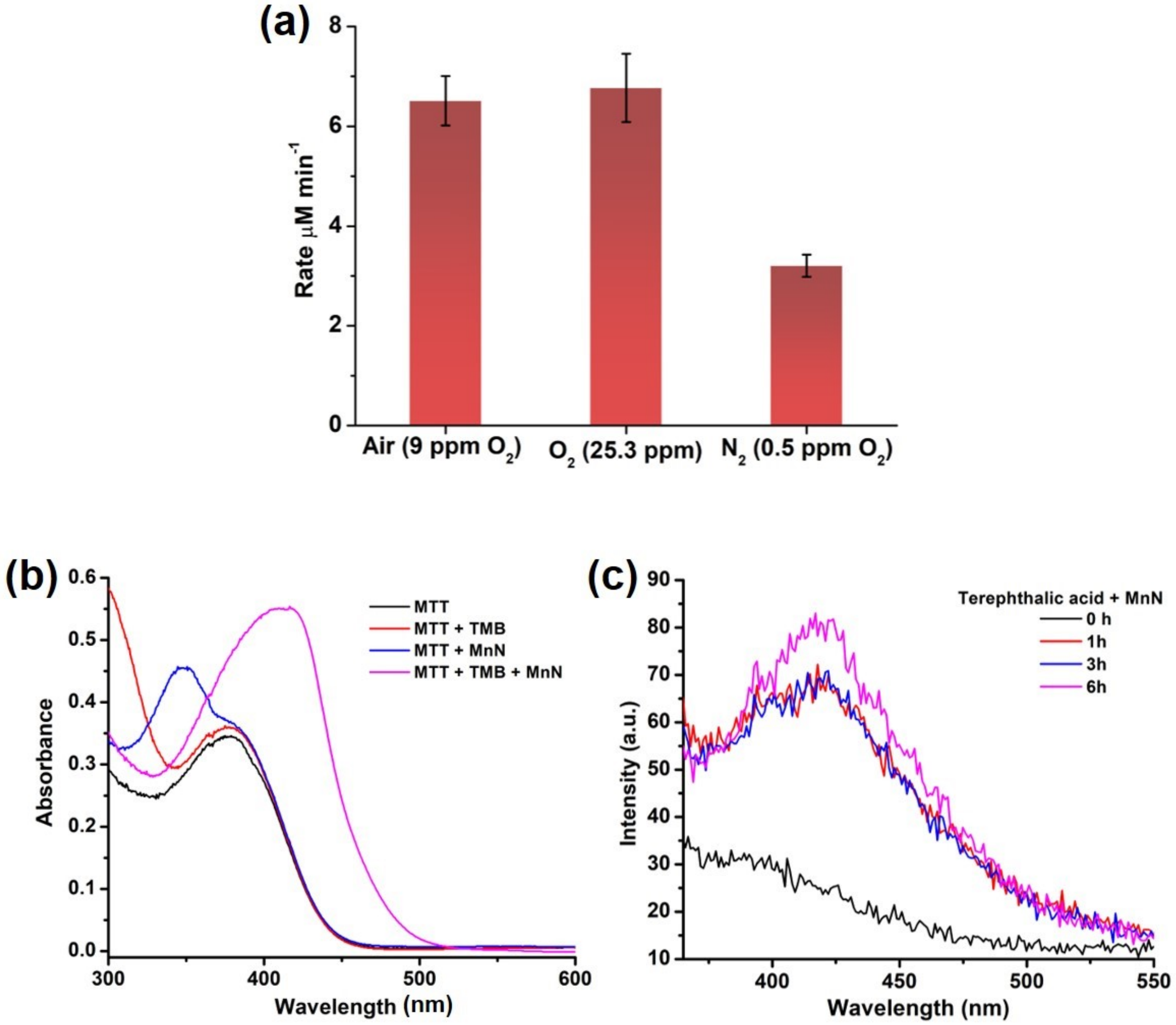

Figure S13. (a) Comparison of oxidase-like activity of MnN in the normal air (9 ppm O_2), O_2-deprived (0.5 ppm O_2), pure O_2 purged conditions (25.3 ppm). (b) UV-vis spectra of different reaction mixtures containing MTT used for probing the formation of superoxide anion radical. The appearance of no signal at 570 nm confirms that superoxide anion radicals are not formed during the reaction. (c) Fluorescence spectra of the detection of hydroxyl radicals by terephthalic acid assay.

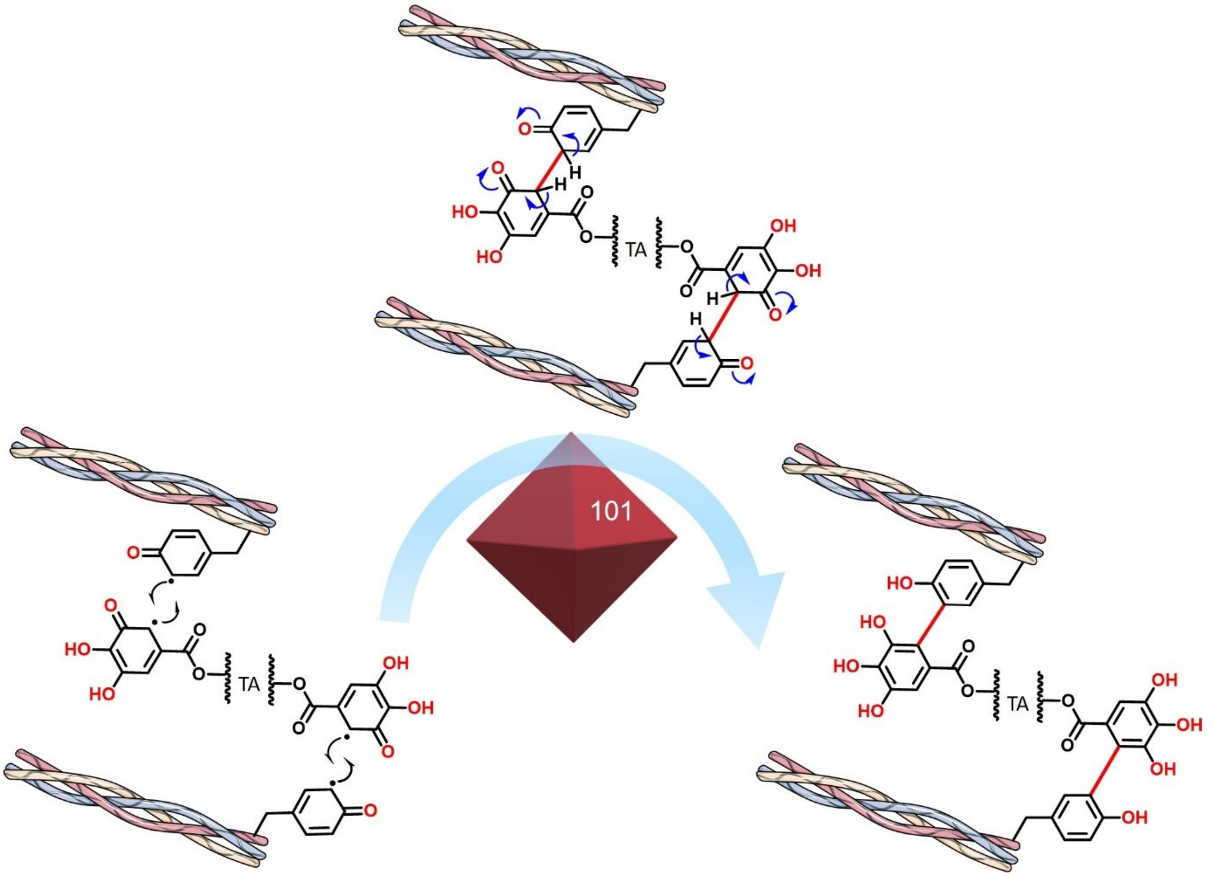

Figure S14. Mechanism showing the covalent crosslinking of collagen as a result of oxidase-like activity of MnN. Note that the covalent crosslinks can also form between a tyrosyl residue of collagen and TA from one side and a Schiff base or Michael adduct from the other side.

References

[1] H. Jiang, T. Zhao, C. Yan, J. Ma, C. Li, *Nanoscale*, 2010, **2**, 2195.

[2] N. Rajan, J. Habermehl, M.-F. Coté, C. J. Doillon, D. Mantovani, *Nat. Protoc.*, 2006, **1**, 2753.

[3] G. Chandrakasan, D. A. Torchia, K. A. Piez, *J. Biol. Chem.*, 1976, **251**, 6062.

[4] J. F. Woessner, *Arch. Biochem. Biophys.*, 1961, **93**, 440.

[5] A.-M. Lawrence, H. U. S. Besir, *J. Vis. Exp.,* 2009.
